# The risk‐benefit ratio of Covid‐19 vaccines: Publication policy by retraction does nothing to improve it

**DOI:** 10.1002/ctd2.35

**Published:** 2022-02-25

**Authors:** Harald Walach, Rainer J. Klement, Wouter Aukema

**Affiliations:** ^1^ Change Health Science Institute Berlin Germany; ^2^ Next Society Institute Kazimieras Simonavicius University Vilnius Lithuania; ^3^ Department of Radiation Oncology Leopoldina Hospital Schweinfurt Germany; ^4^ Independent Data Analyst Hoenderloo Netherlands

On June 24, 2021 we published a risk‐benefit analysis of COVID‐19 vaccines, admittedly based on a shaky database. This analysis resulted in the insight, that very likely for three deaths prevented by vaccination we will have to accept that about two people die as a consequence of these vaccinations.^1^ This analysis led to editors’ threatening to withdraw, and the journal indeed retracted the publication. After a thorough re‐review, it was republished.^2^, ^3^ The database we based our analysis on was a large naturalistic study of the BioNTech vaccine in Israel.^4^ This was the only study at the time that allowed for a direct estimation of an absolute risk reduction (ARR) in mortality. Admittedly, the ARR estimate was only available for a short observation period of 4 weeks after the first vaccine dose, a point raised by critics. One might have wanted a longer observation period to bring out the benefit of vaccinations more clearly, and our estimate of a number needed to vaccinate (NNV) of 16 000 to prevent one death might have been overly conservative.

The recently published 6‐month interim report of the BioNTech‐regulatory clinical trial now covers a period long enough to let us look at this risk benefit ratio once again.^5^ In Table S4 of this publication,^5^ 14 deaths are reported in the placebo group (*n* = 21 921) and 15 in the vaccination group (*n* = 21 926). Among them, two deaths in the placebo‐group were attributed to COVID‐19, and one in the vaccination group was attributed to COVID‐19 pneumonia. This leads to an ARR = 4.56 × 10^–5^, and conversely to an NNV = 1/ARR = 21 916 to prevent one death by COVID‐19. This shows that our original estimate was not so far off the mark.

The most recent safety report of the German Paul Ehrlich Institute (PEI) that covers all reported side effects since the vaccination campaign began (27 December 2020 until 30 November 2021; https://www.pei.de/SharedDocs/Downloads/DE/newsroom/dossiers/sicherheitsberichte/sicherheitsbericht‐27‐12‐20‐bis‐30‐11‐21.pdf?__blob=publicationFile&v=9 accessed 15^th^ Jan 2022) reports 0.02 deaths per 1000 BioNTech vaccinations or 2 per 100 000 vaccinations. We had gleaned four mortality cases per 100 000 vaccinations (all vaccines) from the Dutch phamacovigilance database LAREB.^3^ Using the data of Thomas et al.,^5^ a liberal NNV = 20 000, we can calculate that by 100 000 vaccinations we save five lives. Using the PEI pharmacovigilance report for the same product, we see that these 100 000 vaccinations are associated with two deaths, while using the LAREB database back in June 2021, they were associated with four deaths across all vaccines and are associated with two deaths in the most recent reports concerning the BioNTech vaccine (www.lareb.nl/coronameldingen accessed 17 January 2022). In other words, as we vaccinate 100 000 persons, we might save five lives but risk two to four deaths. This does not take into account the fact that

1. These passive pharmacovigilance data, different from the active reporting system of trials and observations, are notorious for underestimating casualties and side effects.^6^
2. Other severe side effects occur, such as myocarditis in young males who according to a recent study suffer a 13.6 fold risk of myocarditis.^7^

Thus, we have novel and worrying data that confirm the analysis we published in summer 2021, urging us to repeat our call for an installment of a European‐wide active monitoring system that documents the safety and efficacy of COVID‐19 vaccines long‐term and for a rational public debate about the risk‐benefit ratio of these novel vaccines.

## CONFLICT OF INTEREST

The authors declare that there is no conflict of interest that could be perceived as prejudicing the impartiality of the research reported.
